# Severity-Dependent Modulation of Red Blood Cell Aging Patterns in Preeclampsia: Insights from Calorimetry and Atomic Force Microscopy

**DOI:** 10.3390/ijms27083633

**Published:** 2026-04-18

**Authors:** Svetla Todinova, Velichka Strijkova, Ariana Langari, Ina Giosheva, Emil Gartchev, Vesela Katrova, Alexey Savov, Sashka Krumova, Tania Pencheva

**Affiliations:** 1 Institute of Biophysics and Biomedical Engineering, Bulgarian Academy of Sciences, “Acad. G. Bontchev” Str., Bl. 21, 1113 Sofia, Bulgaria; arianalangari@abv.bg (A.L.); ina_gi@abv.bg (I.G.); sashka.b.krumova@gmail.com (S.K.); tania.pencheva@biomed.bas.bg (T.P.); 2 Institute of Optical Materials and Technologies “Acad. Yordan Malinovski”, Bulgarian Academy of Sciences, “Acad. G. Bontchev” Str., Bl. 109, 1113 Sofia, Bulgaria; vily@iomt.bas.bg (V.S.); vlozanova@iomt.bas.bg (V.K.); 3 Medical Center Markovs, 1404 Sofia, Bulgaria; 4 University Obstetrics and Gynecology Hospital “Maichin Dom”, 1431 Sofia, Bulgaria; egartt@gmail.com (E.G.); alexey.savov@abv.bg (A.S.)

**Keywords:** preeclampsia, red blood cell, differential scanning calorimetry, atomic force microscopy, Band 3, hemoglobin, Young’s modulus

## Abstract

Preeclampsia (PE) is associated with systemic oxidative stress and vascular dysfunction, yet its effects on red blood cell (RBC) stability and mechanics remain incompletely understood. Here, we investigate the structural and nanomechanical alterations of RBCs in third-trimester pregnancies complicated by non-severe and severe PE, compared with normotensive controls. RBCs are analyzed using differential scanning calorimetry (DSC) to assess protein thermal stability and atomic force microscopy (AFM) to determine membrane elasticity (Young’s modulus) during in vitro aging. Linear mixed-effects models are applied to evaluate the effects of disease severity, storage time, and their (group × storage time) interaction. DSC reveals that Band 3 and hemoglobin exhibited pronounced destabilization in PE, with severe cases showing earlier and larger reductions in transition temperatures and heat capacities, indicative of disrupted membrane–cytoskeletal interactions. AFM confirms that these molecular changes translate into functional consequences: control and non-severe PE RBCs show physiological softening over time, whereas severe PE RBCs undergo pathological stiffening. Statistical modeling demonstrates strong time, group, and interaction effects for both thermodynamic and mechanical parameters. Together, these findings identify the Band 3–hemoglobin macrocomplex as a primary target of PE-induced RBC alterations and suggest that combined thermodynamic–nanomechanical profiling can serve as a sensitive approach to detect early subclinical RBC damage not detectable by routine hematological tests.

## 1. Introduction

Preeclampsia (PE) is a complex, multisystem disorder of pregnancy characterized by new-onset hypertension and proteinuria after 20 weeks of gestation, remaining one of the leading causes of maternal and perinatal morbidity and mortality worldwide [1]. Globally, PE affects approximately 2–8% of all pregnancies, underscoring its substantial clinical impact [2]. Hypertensive disorders of pregnancy, including preeclampsia and eclampsia, are responsible for an estimated ~16% of maternal deaths (~42,000 annually) and contribute significantly to adverse perinatal outcomes (WHO, 2025) [3], as well as to over 2 million disability-adjusted life years (DALYs) globally each year (WHO, 2025; GBD 2021) [4]. Preeclampsia is increasingly recognized as an independent risk factor for future cardiovascular disease, with affected women exhibiting a higher incidence of hypertension, ischemic heart disease, and stroke later in life [5].

Although the exact etiology of PE is multifactorial and incompletely understood, placental dysfunction, particularly inadequate trophoblastic invasion and incomplete remodeling of the spiral arteries, is recognized as a central initiating event. The resulting placental hypoxia triggers oxidative stress, inflammation, and systemic endothelial dysfunction, all of which contribute to the clinical manifestations of PE [6,7].

Red blood cells (RBCs) play a crucial role in oxygen transport and vascular homeostasis. In normal pregnancy, adaptive changes in RBC morphology, deformability, and antioxidant capacity facilitate efficient oxygen delivery to meet the increased metabolic demands of the mother and fetus. However, in preeclampsia, these adaptations are often compromised. Hypoxia and oxidative stress can induce structural and functional modifications in erythrocyte membranes, hemoglobin molecules, and cytoskeletal components [8]. Such changes can impair RBC deformability and oxygen-carrying efficiency, thereby exacerbating tissue hypoxia and vascular dysfunction, forming a vicious cycle that further aggravates disease progression [9].

Recent findings indicate that RBCs in PE exhibit accelerated aging-like features, including membrane lesions, cytoskeletal remodeling, reduced fluidity, and increased susceptibility to hemolysis [10,11]. The normal erythrocyte lifespan is shortened, resulting in the accumulation of senescent cells with reduced deformability and oxygen-carrying capacity. At the molecular level, protein denaturation, aggregation, and membrane remodeling were proposed as key contributors to these changes [12]. Understanding the biophysical and thermodynamic basis of these alterations is therefore essential for elucidating the mechanisms underlying erythrocyte dysfunction in preeclampsia.

The thermal stability of erythrocyte components provides valuable insight into the structural and functional integrity of RBCs under physiological and pathological conditions. Differential scanning calorimetry (DSC) is a highly sensitive technique that detects the thermal unfolding of membrane and cytoskeletal proteins, thereby revealing subtle conformational changes associated with cellular aging and disease states [13,14,15]. In PE, oxidative stress and membrane remodeling are expected to alter protein stability, yet the thermodynamic manifestations of these disturbances remain insufficiently characterized.

Beyond thermodynamic stability, the mechanical properties of RBCs are central to their ability to traverse the microvasculature. Atomic force microscopy (AFM) enables nanoscale quantification of cell stiffness by measuring Young’s modulus (Ea), providing direct insight into membrane rigidity, cytoskeletal remodeling, and the biomechanical consequences of disease [16,17]. While alternative nanoscale techniques such as optical tweezers provide highly sensitive measurements and valuable quantitative information on cell deformability, viscoelastic response, and force–extension behavior in whole-cell suspensions [18,19], AFM offers distinct advantages. In particular, AFM combines high-resolution topographical imaging with direct, localized mechanical probing, enabling precise spatial mapping of membrane stiffness across specific regions of the cell surface. Unlike optical tweezers, which primarily assess global mechanical responses, AFM allows localized nanomechanical characterization and facilitates direct correlation between mechanical properties and surface morphology.

Taken together, DSC and AFM offer powerful means of characterizing the thermodynamic stability and mechanical resilience of RBCs under pathological conditions such as PE.

In this study, we investigate how PE affects the thermodynamic and nanomechanical properties of red blood cells, with particular emphasis on disease severity and cell aging. By comparing RBCs from healthy pregnant women and patients with non-severe and severe PE, we aim to identify characteristic biophysical signatures that could elucidate the mechanisms underlying erythrocyte destabilization, impaired oxygen transport, and accelerated cellular senescence in PE.

## 2. Results

### 2.1. Patients’ Characteristics

The clinical characteristics and hematological parameters of the study population are summarized in Table 1. The cohort comprised 28 women with PE, including 20 non-severe and 8 severe cases, classified according to established clinical criteria based on blood pressure levels, proteinuria, and the presence of end-organ complications (see Materials and Methods), along with 14 normotensive pregnant controls (PC).

Women with PE were slightly older than controls, with median maternal age correlating with disease severity (Table 1). As expected, both systolic and diastolic blood pressures were markedly elevated in PE patients compared to PC, with the highest values observed in the severe PE subgroup.

Gestational age (GA) at delivery was progressively reduced with increasing PE severity. Normotensive pregnant women delivered at term, whereas women with non-severe PE delivered earlier (37 [31.8; 38] weeks), and those with severe PE experienced markedly preterm delivery (29 [28; 29.5] weeks). This was reflected in neonatal outcomes, with a substantial decrease in birth weight from controls (3330 [3155; 3457.5] g) to non-severe PE (2770 [2130; 3080] g) and severe PE (1060 [852.5; 1425] g).

Proteinuria was detected in both PE groups and was more pronounced in severe PE. Median 24 h urinary protein excretion was 482 ± 320 mg, observed in 8 patients (40%) in the non-severe PE group, and increased to 1736.6 ± 320 mg in the severe PE group, where proteinuria was present in 6 of all 7 patients (Table 1).

Analysis of hematological parameters demonstrated that median RBC count, hemoglobin (Hb), hematocrit (Ht), and RBC indices remained within pregnancy-specific reference ranges across all groups. Despite these overall normal values, considerable inter-individual variability was observed, particularly among women with PE. Median RBC counts and Hb concentrations were comparable between groups (Table 1). Nevertheless, a substantial proportion of both PC and PE patients exhibited RBC counts below the lower reference limit (42% in PC, 45% in the non-severe PE group, and 62% in the severe PE group), accompanied by reduced Hb levels (28%, 50% and 38%, respectively), consistent with mild to moderate anemia.

Mean corpuscular indices, i.e., mean corpuscular volume (MCV), mean corpuscular hemoglobin (MCH), and mean corpuscular hemoglobin concentration (MCHC), were within pregnancy-specific reference ranges and did not differ substantially between controls and PE groups (Table 1), with a slight increase in MCV observed in severe PE. Red cell distribution width (RDW) values were comparable across groups, showing greater variability in non-severe PE and slightly lower median values in severe PE.

### 2.2. DSC Profiles of RBCs Isolated from Pregnant Women in the Third Trimester and from Preeclampsia Patients

**Figure 1:**
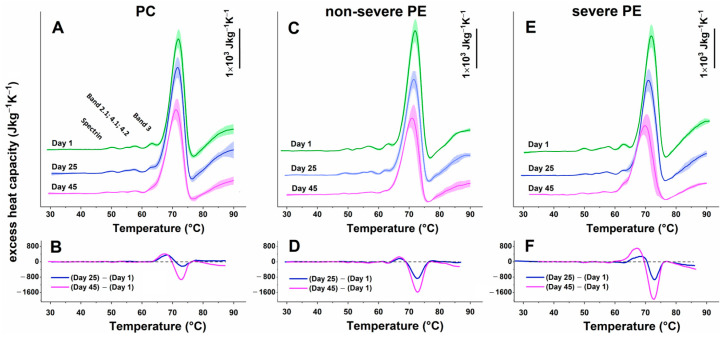
Average DSC profiles (± standard deviation) of RBCs derived from pregnant controls in the third trimester (**A**), patients with non-severe PE (**C**), and patients with severe PE (**E**). The data are obtained from freshly isolated (day 1) RBCs and cells stored for 25 and 45 days. For clarity of presentation, the thermograms in (**A**,**C**,**E**) are vertically displaced along the Y-axis; a calibration marker (1 × 10^3^ J·kg^−1^·K^−1^) is included as a reference scale. The difference curves in (**B**,**D**,**F**) are obtained by subtracting the calorimetric profile of fresh RBC (Day 1) from the corresponding curves obtained after 25 or 45 days of storage for each sample. All values are expressed in SI units (J·kg^−1^K^−1^).

The average DSC thermogram of freshly isolated RBCs from normotensive pregnant women in the third trimester (Figure 1A) exhibited multiple sequential thermal transitions characteristic of RBC protein denaturation. These included low-temperature transitions associated with the denaturation of cytoskeletal proteins, i.e., spectrin (T_m_^spectrin^, ~50 °C), Bands 2.1/4.1/4.2 (T_m_^Band2–4^, ~57 °C), and the cytoplasmic domain of Band 3 protein (T_m_^Band3^, ~63 °C), followed by a dominant endothermic peak corresponding to Hb denaturation at ~72 °C and a subsequent exothermic event at ~77 °C, as previously reported for healthy donors [20].

The thermograms of freshly obtained RBCs from patients with PE did not differ markedly from those of the control group. However, there was a slight downshift in the denaturation temperature of T_m_^Band2–4^ in the non-severe PE group (Figure 1, Table 2), suggesting subtle early cytoskeletal alterations.

More pronounced group- and severity-dependent differences emerged during in vitro storage, indicating that, in addition to physiological aging, preeclampsia exerts a distinct influence on the thermodynamic behavior of RBC components.

In Table 2, values labeled with different lowercase letters denote statistically significant differences between storage days within the same group, whereas different uppercase letters indicate significant differences between groups at the same storage day.

The denaturation temperature of spectrin exhibited a decreasing tendency during storage in all groups. However, this decline reached statistical significance only at day 45 in the control and severe PE groups, whereas no statistically significant differences were observed between days 25 and 45 in the non-severe PE group. Overall, the extent of the observed changes was comparable across groups. These findings suggest that spectrin thermal stability is influenced primarily by storage-related effects rather than disease-specific conditions, although a modest group effect was detected. Linear mixed-effects analysis confirmed a strong main effect of storage time (denoted as Day) on T_m_^spectrin^ (η^2^_p_ = 0.673), indicating that storage duration was the dominant factor affecting protein thermal stability. A significant effect of Group was also observed (η^2^_p_ = 0.218), whereas the Day × Group interaction was not significant (η^2^_p_ = 0.022) (Table 3).

T_m_^Band2–4^ remained relatively stable throughout the storage in the control and non-severe PE groups, with no consistent statistically significant changes across time points. In the severe PE group, a modest but statistically significant decrease was observed at day 45 compared to earlier storage times, although the overall effect sizes were small (η^2^_p_ = 0.037–0.098), indicating limited contribution to global thermodynamic changes (Table 3).

The thermal transition associated with the Band 3 cytoplasmic domain displayed a clear pathology-dependent pattern. In controls, T_m_^Band3^ remained essentially unchanged over time, with no statistically significant differences between storage days, indicating preserved thermal stability. In contrast, the non-severe PE group showed a progressive and statistically significant decrease in T_m_^Band3^ (≈1.4 °C by day 45), accompanied by a marked reduction in its excess heat capacity (c_P_^Band3^), suggesting reduced conformational stability (Table 2). The severe PE group exhibited a more complex behavior. T_m_^Band3^ decreased modestly between Day 1 and Day 25, with no further significant change at Day 45, suggesting an early shift followed by stabilization. In parallel, c_P_^Band3^ exhibited a biphasic pattern, i.e., an initial reduction followed by a pronounced increase at day 45, suggesting altered membrane protein interactions and possible reorganization of Band 3–cytoskeletal coupling in advanced disease.

Statistical analysis using linear mixed-effects models revealed significant effects of Day and Group on both T_m_^Band3^ and c_P_^Band3^, with significant Day × Group interactions (T_m_^Band3^: F = 3.82, η^2^_p_ = 0.115; and c_P_^Band3^: F = 7.21, η^2^_p_ = 0.198, respectively). According to conventional benchmarks for interpreting partial eta squared, these correspond to moderate-to-large and large effect sizes, respectively, indicating that aging and pathology act synergistically on Band 3 thermal stability (Table 3). The observed interaction effects highlight the increased susceptibility of the RBC membrane–cytoskeleton complex to structural alterations in PE.

Hb thermal stability was also affected in a severity-dependent manner. In the thermograms of the PC group, T_m_^Hb^ and c_P_^Hb^ showed only a subtle decline in the course of storage. A similar trend was evident in non-severe PE, albeit with a more pronounced trend and slightly lower c_P_^Hb^ values compared to the control group (Figure 1C, Table 2). In the severe PE group, however, Hb exhibited the greatest destabilization, with a stronger decline in transition temperature and the steepest decrease in c_P_^Hb^ detected over the ageing period, reflecting substantially reduced thermal stability (Figure 1E, Table 2). Linear mixed-effects modeling confirmed strong Day × Group effects, as well as significant interactions (η^2^_p_ = 0.150 for T_m_^Hb^; η^2^_p_ = 0.142 for c_P_^Hb^, Table 3), indicating that Hb is highly sensitive to both aging and gestational pathology.

#### 2.2.1. Difference Thermograms

To enhance sensitivity and better visualize aging-related structural changes, difference thermograms were generated by subtracting the calorimetric curves recorded on days 25 and 45 from those obtained on day 1 (Figure 1B,D,F). This approach eliminates baseline variability, revealing aging-associated thermodynamic alterations that were not readily apparent in the raw calorimetric data before standard parameter analysis. The resulting difference curves revealed significantly greater thermodynamic shifts in PE samples over time, particularly in the severe PE group. The most prominent alterations occurred in the Band 3 and hemoglobin transition regions, indicating either accelerated or more extensive protein destabilization, as well as membrane-related changes in PE samples.

#### 2.2.2. Time- and Severity-Dependent Effects on RBC Protein Stability

Linear mixed-effects analysis was applied to evaluate the combined fixed effects (PE, ageing trends) with random effects (variation within PC and PE groups) to analyze the obtained dataset.

Linear mixed-effects analysis demonstrated progressive thermodynamic destabilization of RBC proteins during storage, and the magnitude of change depended on both gestational pathology and time (Table 3). While spectrin destabilization predominantly reflected physiological aging, Band 3 and Hb exhibited the strongest severity-related effects and significant interaction effects. These findings support the hypothesis that altered Band 3–Hb interactions represent a central mechanism underlying increased erythrocyte fragility and reduced membrane stability in preeclampsia [10]. The statistical verification of the observed calorimetric deviations indicates that preeclampsia accelerates molecular aging of RBCs, particularly affecting membrane–cytoskeletal coupling and hemoglobin stability, with the most severe alterations observed in advanced disease.

### 2.3. Elastic Properties of RBCs Isolated from Pregnant Women in the Third Trimester and from Preeclampsia Patients

Next, we assessed how these molecular alterations affect the mechanical properties of RBCs using AFM. This analysis enabled us to correlate changes in thermal stability with biomechanical behavior, providing a more complete picture of how preeclampsia, especially in its severe form, impacts RBC structure and deformability, with potential consequences for microcirculatory function.

**Figure 2:**
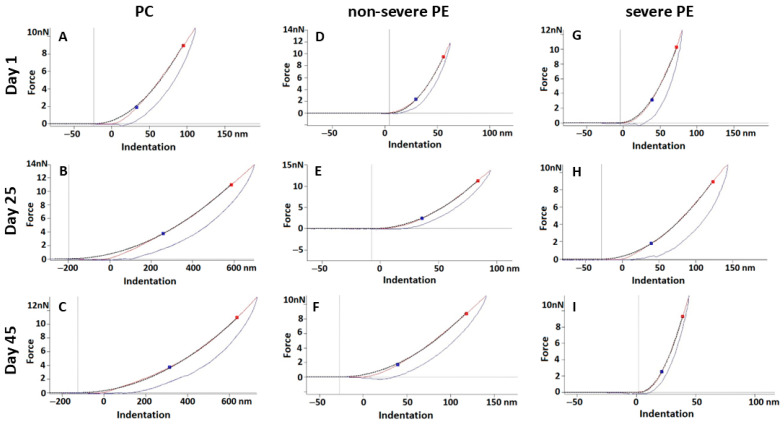
Representative force–indentation curves obtained from RBC samples of control (PC; (**A**–**C**)), non-severe PE (**D**–**F**), and severe PE (**G**–**I**) groups at different stages of cell aging.

The representative force–indentation curves presented in Figure 2 depict the mechanical behavior of RBC membranes, revealing group- and aging-dependent differences in deformation profiles between control and PE samples.

In the present study, RBCs from healthy pregnant women in the third trimester displayed a significantly higher apparent Young’s modulus (Ea ≈ 1.53 ± 0.3 MPa) compared to healthy pregnant controls in the first trimester (Ea ≈ 1.12 ± 0.3 MPa) for freshly isolated cells, and of non-pregnant controls (Ea ≈ 0.94 ± 0.2 MPa), as reported in our previous investigation [20]. This comparison indicated a consistent increase in RBC membrane stiffness during pregnancy, which appears to be further modulated by disease status and cellular aging.

**Figure 3:**
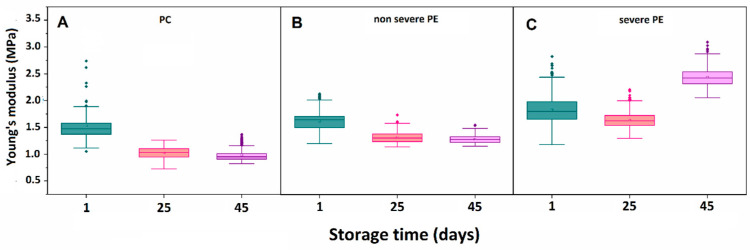
Young’s modulus determined during the aging of RBCs isolated from healthy pregnant women in the third trimester of pregnancy (**A**), patients with non-severe (**B**) and severe (**C**) preeclampsia.

During storage, RBC elasticity displayed a clear time- and disease-dependent modulation (Table 4, Figure 3). In the PC group, Ea decreased significantly from 1.46 MPa (at day 1) to 1.03 MPa (at day 25, *p* ≤ 0.05) and remained stable at 0.95 MPa (at day 45), reflecting progressive membrane softening with aging. However, it should be noted, that the confidence intervals at days 25 and 45 are relatively wide, likely reflecting increased variability in the measured values (Table 4).

In RBCs from non-severe PE patients, Ea values were consistently higher than those of controls throughout the aging period, although they followed a similar decreasing trend (Figure 3B, Table 2). Ea declined from 1.64 MPa (at day 1) to 1.31 MPa (at day 25) and 1.27 MPa (at day 45), representing a smaller relative decrease (~20% vs. ~30% in PC), with values remaining significantly elevated compared to PC at day 25 and day 45 (*p* ≤ 0.05). This suggests partial preservation of membrane elasticity during storage despite aging. The relatively narrow confidence intervals across all time points indicate low variability and good consistency of the measurements within this group.

By contrast, RBCs from patients with severe PE exhibited a distinct biphasic mechanical behavior (Figure 3C). At baseline (day 1), Ea (1.81 MPa) was already significantly higher than both PC and non-severe PE (*p* < 0.05), reflecting increased membrane stiffness. Although Ea decreased slightly by day 25 to 1.63 MPa, it increased markedly by day 45 to 2.43 MPa, surpassing both its own baseline and PC values (*p* < 0.05). The confidence intervals in this group were wider at the earlier time points, but became notably narrower at day 45, suggesting increased homogeneity of the mechanical response in aged RBCs under severe disease conditions. This pattern suggests profound remodeling of the cytoskeleton and membrane mechanics during advanced stages of cell aging in severe PE, distinguishing it from both controls and non-severe PE (Figure 3, Table 4).

These observations were supported by a linear mixed-effects model evaluating the effects of storage time (Day), disease severity (Group), and their interaction on Ea (Table 5). Significant main effects were detected for Group (*p* < 0.001, η^2^_p_ = 0.498), reflecting overall differences in RBC stiffness among clinical groups, and for Day (*p* < 0.001, η^2^_p_ = 0.871), confirming progressive softening during storage. Importantly, a significant Day × Group interaction (*p* < 0.001, η^2^_p_ = 0.729) indicated that the temporal changes in Ea differed depending on disease severity, with severe PE RBCs exhibiting both elevated baseline stiffness and a pronounced increase at later storage, in contrast to PC and non-severe PE (Table 5).

**Figure 4:**
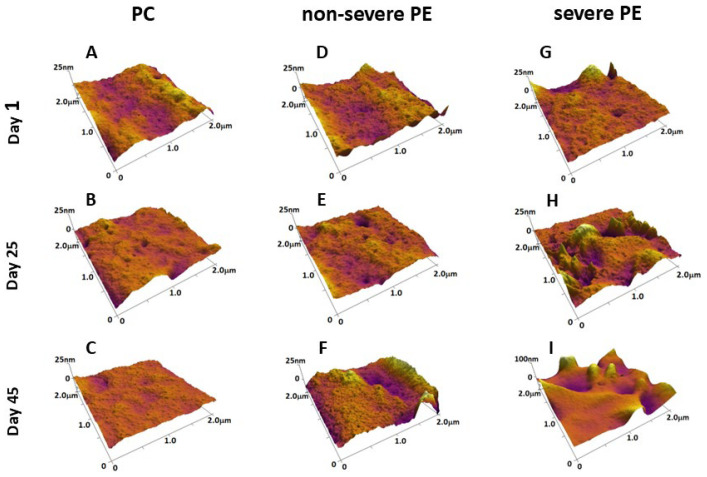
Selected 3D AFM images, representing the changes in membrane surface topology of RBCs isolated from pregnant controls (**A**–**C**), patients with non-severe (**D**–**F**), and severe (**G**–**I**) PE, as a function of cell aging. The scanned area is 2 × 2 µm^2^.

To further characterize nanoscale membrane alterations, we performed a quantitative analysis of RBC surface roughness using AFM topographical images (Figure 4). Root-mean-square (R_rms_) roughness was evaluated to assess changes in membrane surface topology associated with disease state and cell aging. In our previous work [10], we demonstrated that the membrane of fresh PE RBCs differed significantly from that of healthy controls by the presence of invaginations and protrusions, accompanied by increased R_rms_ values. Moreover, PE RBC aging resulted in more pronounced surface features, with a progressive increase in R_rms_ values, whereas in control cells, R_rms_ decreased approximately linearly over time. However, in that study, PE cases were not stratified according to disease severity.

In the present work, we extend these observations by analyzing RBC roughness separately in non-severe and severe PE groups, allowing for a more detailed assessment of severity-dependent differences in membrane surface topology during cell aging. The results indicate that membrane roughness increases with disease severity and aging, supporting the notion that structural membrane alterations are more pronounced in severe PE and are consistent with the mechanical changes observed in the AFM-based elasticity measurements.

In the current dataset, control samples exhibited relatively smooth membrane surfaces, with R_rms_ decreasing gradually during storage (from 3.82 ± 0.7 to 2.23 ± 0.6 nm), consistent with progressive membrane remodeling and vesiculation (Figure 4A–C). In contrast, RBCs from non-severe PE patients showed a moderate increase in roughness compared to controls across all time points (e.g., 4.18 ± 0.8–8.24 ± 2.3 nm), indicating subtle surface irregularities that persist with aging (Figure 4D–F).

RBCs from patients with severe PE showed significantly elevated roughness values, particularly at later stages of storage, where R_rms_ increased significantly (from 4.39 ± 0.9 to 11.24 ± 7.1 nm). This increase was accompanied by pronounced surface heterogeneity, suggesting substantial disruption of membrane organization and cytoskeletal integrity.

### 2.4. Advanced Oxidation Protein Products in Plasma and Their Influence on RBC Alterations

Oxidative stress is associated with the development and progression of pregnancy and mainly with its complications, such as PE. As RBCs are continuously exposed to the plasma environment and are highly susceptible to oxidative damage, we assessed advanced oxidation protein products (AOPP) as indicators of the oxidative milieu surrounding circulating RBCs.

Our results showed that AOPP levels in normotensive pregnant controls were slightly higher than values described for first-trimester pregnancy [21] (Table 6). This modest elevation is consistent with the physiological increase in oxidative processes during pregnancy and suggests that RBCs in uncomplicated pregnancies are exposed to a relatively controlled oxidative environment. In non-severe PE, AOPP levels were elevated compared to controls, although the difference did not reach statistical significance, indicating a trend toward increased oxidative exposure that may begin to affect RBCs. In contrast, severe PE was characterized by a pronounced and statistically significant increase in AOPP levels, indicating substantially greater oxidative loading of the plasma.

## 3. Discussion

In this study, we combine DSC and AFM to gain insight into the red blood cell alterations in preeclampsia. While previous research has documented oxidative stress, impaired deformability, and membrane lesions in PE [10,22], the integration of thermodynamic and nanomechanical analyses performed here provides an additional viewpoint: DSC reveals molecular-level instability of membrane and cytoskeletal proteins, while AFM detects the resulting consequences at the nanoscale, including changes in stiffness and mechanical resilience.

We demonstrate time- and severity-dependent shifts in the thermal transitions of Band 3 and hemoglobin, accompanied by increased membrane stiffness. These data establish a direct association between protein destabilization and biomechanical dysfunction, processes rarely investigated in parallel in PE studies. These abnormalities intensify during in vitro aging, indicating that RBCs in PE are intrinsically compromised and deteriorate more rapidly than those obtained from healthy pregnancies.

Distinct thermodynamic signatures (T_m_ and c_P_^ex^ shifts), together with AFM-derived mechanical profiles, allow multidimensional characterization of RBC damage and may serve as biophysical indicators of disease severity. The pronounced alterations observed in severe PE underscore the contribution of RBC fragility and impaired microcirculatory flow [10,22] to the systemic hypoxia and vascular dysfunction characteristic of this disorder [9,23].

### 3.1. RBC Thermodynamic Alteration in PE

In the last few decades, DSC has gained recognition in the studies of disease diagnostics and body fitness based on the thermal properties of biofluids. Recent studies have demonstrated its applicability in evaluating disease-associated alterations of major plasma and RBC proteins as well as their interactions with drugs [24,25,26].

In this context, our DSC analysis demonstrates that RBC remodeling in PE reflects both accelerated aging and qualitative, disease-specific changes. In particular, in healthy third-trimester pregnancies, gradual decreases in the transition temperature T_m_^spectrin^, in the heat capacities of hemoglobin (c_P_^Hb^) and the cytoplasmic domain of Band 3 protein (c_P_^Band3^) were observed during storage, whereas the transition temperatures T_m_^Hb^ and T_m_^Band3^ remained largely unchanged. A similar trend was observed previously in controls from the first trimester of pregnancy, suggesting that the stability of these proteins is not strongly influenced by gestational stage [27]. The preservation of hemoglobin transition contrasts with observations in healthy non-pregnant controls, which exhibit progressive destabilization of Hb transition with age [27]. These data imply the presence of compensatory protective mechanisms during pregnancy against oxidative stress and structural compromise that preserve hemoglobin structure and function. It is well-known that normal pregnancy involves increased erythrocyte catalase (CAT) activity (roughly 3-fold) to combat higher oxidative stress across all three trimesters, preserving hemoglobin structure and function. However, in pre-eclamptic women, this compensatory mechanism fails, leading to lower catalase activity compared to healthy controls [28,29,30]. In line with this, in non-severe PE, we detected greater thermodynamic changes, with Band 3 exhibiting a downward shift in T_m_ and a pronounced decrease in c_P_. Hb transition amplitude declined more rapidly than in PC, consistent with accelerated oxidative remodeling within a pro-inflammatory and pro-oxidative maternal milieu. PE is characterized by intensified systemic inflammation and placental oxidative stress, associated with increased release of syncytiotrophoblast-derived debris, pro-inflammatory cytokines, and anti-angiogenic factors (e.g., sFlt-1), as well as reactive oxygen species into the maternal circulation from the hypoxic placenta [31], which likely collectively contribute to enhanced hemoglobin susceptibility to oxidative modification [32]. T_m_^Hb^ downward shift was stronger for PE than for PC, most likely reflecting faster oxidation of hemoglobin to methemoglobin (metHb). These observations could indicate either that oxidized hemoglobin is intrinsically less thermally stable than functional oxyhemoglobin or that hemoglobin in PE converts more rapidly to metHb, resulting in a higher fraction of less stable metHb at the time of measurement. Previous DSC investigations have shown that the thermal stability of hemoglobin depends on both its redox state and environmental conditions. Under near-physiological conditions (pH 7.4), oxyhemoglobin exhibits a denaturation temperature (approximately 71 °C), whereas metHb, in which iron is oxidized to the ferric state (Fe^3+^), denatures at a lower temperature (around 67 °C) [33]. Michnik et al. further demonstrated that at pH 6.5, Hb denatures at an even lower temperature [34]. This decrease in thermal stability reflects conformational alterations associated with oxidation of the heme iron and changes in intramolecular interactions that reduce overall structural stability.

Severe PE demonstrated a qualitatively distinct thermodynamic signature characterized by: (i) earlier downshifts of T_m_^Hb^ and T_m_^Band3^; (ii) progressive reductions in c_P_^Hb^; and (iii) specific late increases in c_P_^Band3^, suggesting an altered behavior of Band 3 protein. Consequently, the reduction in T_m_^Hb^ indicates destabilization of the Band 3–hemoglobin macrocomplex, an essential structural and functional interface linking hemoglobin to the spectrin–ankyrin cytoskeleton [35]. The concomitant alterations of T_m_ and c_P_^ex^ of Hb and Band 3 protein suggest selective vulnerability of the Band 3–Hb complex in severe PE. In contrast, spectrin and Bands 2–4 remained thermodynamically stable (comparable to controls), underscoring that the principal thermodynamic disturbance is concentrated within the Hb - Band 3 protein macrocomplex. This observation is consistent with previous studies highlighting the central role of Band 3 in cytoskeletal connectivity, thereby coordinating oxygen transport, metabolic regulation, and structural integrity [36,37]. Disruption of this interface under disease conditions can propagate cytoskeletal tension imbalances, compromise membrane resilience, and predispose erythrocytes to premature senescence.

Oxidative stress provides a plausible explanation. Oxidized and/or denatured hemoglobin can copolymerize with the cytoplasmic domain of Band 3, promoting Band 3 clustering that facilitates autoantibody binding and subsequent immune-mediated clearance of erythrocytes [38]. Enhanced oxidative damage also promotes Band 3 oligomerization and triggers irreversible membrane alterations, shifting the membrane toward a less stable thermodynamic state. In agreement, Akoev et al. demonstrated that oxidative stress reduces transition temperatures and destabilizes membrane protein thermal transitions due to oxidative aggregation of cytoskeletal components [39].

It should be noted that, in the present study, oxidative stress exposure of RBCs was assessed indirectly through plasma AOPP concentration, which reflects the systemic oxidative milieu to which RBCs are exposed. Although this approach does not provide direct information on RBC-specific oxidative stress markers, the elevated AOPP levels observed in PE plasma, mainly in severe cases, are consistent with the thermodynamic destabilization of the Band 3–hemoglobin macrocomplex. These findings support the interpretation that oxidative stress contributes to the observed RBC alterations. The application of additional oxidative markers remains an important goal for future research.

All together, these findings indicate that severe PE induces qualitative remodeling of RBCs rather than merely accelerating physiological aging, with the Band 3–Hb macrocomplex emerging as a primary thermodynamic and structural target.

### 3.2. RBC Nanomechanical Changes in PE

AFM has similarly advanced the field of biomedicine by providing high-resolution insights into the morphological and nanomechanical features of cells. Recent studies demonstrate its growing importance in disease-related investigations, where it enables the detection of subtle mechanical alterations associated with pathological states [40,41]. In the present work, AFM analysis complements the thermodynamic data, revealing a progression from adaptive to pathological mechanical remodeling across the different groups upon cell ageing.

Freshly isolated RBCs from PC exhibited elevated baseline stiffness compared with first-trimester or non-pregnant controls [20]. This increased stiffness likely reflects reversible structural stiffening due to cumulative oxidative stress, hormonal modulation of membrane composition, and/or low-grade inflammation characteristic of late gestation, rather than irreversible damage.

During in vitro aging of RBCs from third-trimester controls, they progressively softened, as indicated by decreases in Young’s modulus (E_a_). This pattern contrasts with our previous observations in first-trimester pregnancies, where RBCs stiffened along the aging path [20]. A plausible interpretation is that the late-gestation softening represents a physiological cytoskeletal adaptation that enhances RBC deformability and supports microvascular perfusion under the high cardiac output, increased plasma volume, and reduced systemic vascular resistance typical of advanced pregnancy. In contrast, the stiffening observed during early gestation may reflect adaptive structural alteration of the RBC membrane in response to the initial hemodynamic and oxidative changes accompanying the onset of pregnancy [42,43,44,45]. Together, the elevated baseline stiffness and progressive softening indicate that RBCs in late gestation maintain a balance between structural resilience and adaptive deformability.

Non-severe PE RBCs exhibited higher baseline stiffness than PC, consistent with increased oxidative stress and membrane protein modification. Despite this elevated rigidity, a pattern of gradual softening during aging persisted, suggesting that cytoskeletal relaxation and dynamic membrane remodeling remain functional. This indicates that RBCs in non-severe PE retain partial adaptive capacity, allowing mechanical adjustment to oxidative and inflammatory challenges. These observations align with prior evidence showing that moderate oxidative modification of membrane proteins can stiffen RBCs while preserving dynamic remodeling capabilities [32,46]. Moreover, even non-severe PE is associated with systemic oxidative stress secondary to placental hypoxia and inflammatory activation [47].

Severe PE RBCs, in contrast, demonstrated progressive and marked stiffening over time, culminating in substantial increases in Young’s modulus by day 45. This mechanical response diverges from both PC and non-severe PE and indicates pathological rigidification rather than accelerated physiological aging. Elevated c_P_^Band3^ during late aging suggests abnormal membrane protein reorganization rather than simple unfolding. This stiffening likely results from oxidative cross-linking of spectrin and Band 3, Band 3 clustering and aggregation, enhanced cytoskeleton–membrane anchoring, lipid peroxidation reducing bilayer fluidity, and impaired ATP-dependent cytoskeletal remodeling [48,49]. Such structural changes reduce RBC deformability, increase vascular resistance, and likely contribute to impaired placental perfusion observed in severe PE.

AFM-based roughness analysis provides complementary insight into nanoscale membrane remodeling during RBC aging. Under physiological conditions, membrane aging is typically associated with progressive surface smoothing, a process linked to vesiculation, the loss of membrane microdomains, and adenosine triphosphate-dependent alteration of the membrane-skeleton properties. This behavior has been described by Girasole et al. [17,50], who reported a reduction in membrane roughness as a hallmark of normal erythrocyte aging. In contrast, under pathological conditions such as preeclampsia, this process appears to be fundamentally altered, leading to increased surface roughness with aging, indicative of structural deterioration. Elevated roughness can be attributed to disrupted membrane–cytoskeleton interactions, oxidative damage to lipids and proteins, or impaired vesiculation. Consistent with this, our previous findings [10] demonstrated that oxidative stress (in particular H_2_O_2_ exposure) induces progressive membrane defects, suggesting compromised antioxidant defense in PE RBCs.

### 3.3. Statistical Analyses

Statistical modeling supported the conclusions drawn so far. Time effects were significant across groups, reflecting aging-driven remodeling, while Day × Group interaction effects for T_m_^Hb^ and T_m_^Band3^ values confirmed disease-specific destabilization. No significant Group effect was observed for T_m_^spectrin^, underscoring the selective vulnerability of the Band 3–Hb macrocomplex. These findings identify hemoglobin and Band 3 as the most sensitive thermodynamic markers of erythrocyte fragility and disease severity. The divergence of T_m_^Hb^ and c_P_^Hb^ in severe PE further reinforces the notion that destabilization arises primarily from perturbations in the membrane–cytoskeletal environment.

Linear mixed-effects analysis also confirmed a significant time effect on Young’s modulus across groups, reflecting aging-dependent mechanical remodeling, alongside significant interaction relations indicating altered stiffness paths in PE. These disease-specific mechanical patterns paralleled the interaction effects detected for T_m_^Hb^ and T_m_^Band3^ transitions in the DSC analysis. The statistical consistency between thermodynamic and nanomechanical parameters supports a coupled disruption of membrane protein stability and RBC mechanical integrity in PE.

### 3.4. Study Limitations

A limitation of this study is the absence of longitudinal follow-up after delivery. Postpartum samples were not collected. Therefore, potential recovery or persistence of the observed alterations in RBC properties following childbirth could not be evaluated.

A second limitation is that the cohort was ethnically homogeneous (European origin), which may limit the generalizability of the findings, as genetic and ethnic background can influence the hematological and biomechanical properties of erythrocytes.

It should also be noted that the relatively small sample size of the severe PE group (*n* = 8) may limit the generalizability of the statistical analysis. Although confidence intervals were included (Table 4) to provide an estimate of variability, the results should be confirmed in larger cohorts.

In addition, oxidative stress assessment was limited to plasma AOPP. While informative of the systemic oxidative environment to which RBCs are exposed, this marker does not capture RBC-specific oxidative damage or antioxidant capacity. The inclusion of additional markers (e.g., TBARS, protein carbonyls, catalase activity) would provide a more comprehensive characterization and should be considered in future studies.

## 4. Materials and Methods

### 4.1. Patient Selection and Ethics Statement

The study was approved by the Ethics Committee of the Institute of Biophysics and Biomedical Engineering, Bulgarian Academy of Sciences (Approval No. 378ND, 26 March 2024) and conducted in accordance with the Declaration of Helsinki (1975, revised 2013). All participants provided written informed consent before enrollment.

The study included 14 normotensive pregnant women in the third trimester (PC; median age 28 (25; 32.8) years) and 28 pregnant women diagnosed with PE, comprising twenty with non-severe PE (median age 30 (27; 34) years) and eight with severe PE (median age 35 (31.8; 36.3) years), admitted to the Hospital of Obstetrics and Gynecology “Maichin Dom,” Medical University of Sofia, and Medical Center Markovs, Sofia. PE diagnosis and severity were established according to current hypertension-in-pregnancy guidelines [Hypertension in Pregnancy: Diagnosis and Management NICE. Available online: https://www.nice.org.uk (Published: 25 June 2019; Last updated: 17 April 2023; accessed on 24 March 2026)].

The diagnosis of PE and its classification into non-severe and severe forms were established according to clinical guidelines adopted in Bulgarian hospitals, consistent with those proposed by the International Society for the Study of Hypertension in Pregnancy (ISSHP) [51]. Non-severe preeclampsia was defined as systolic blood pressure ≥140 mmHg and/or diastolic blood pressure ≥90 mmHg after 20 weeks of gestation, in the absence of severe features. Severe preeclampsia was defined by systolic blood pressure ≥160 mmHg and/or diastolic blood pressure ≥110 mmHg and/or the presence of maternal or fetal complications, including renal impairment, elevated liver enzymes, thrombocytopenia, neurological symptoms, pulmonary edema, or fetal growth restriction. Patient classification in the present cohort was based on blood pressure measurements, proteinuria assessment, gestational age at diagnosis, and the presence of maternal and fetal complications.

Control participants had uncomplicated pregnancies, normal blood pressure, and delivered after 38 weeks of gestation. Exclusion criteria encompassed chronic hypertension, thyroid disorders, diabetes, kidney disease, erythrocytopathies, autoimmune conditions, hyperlipidemia, and fetal malformations.

All participants were of European origin. Therefore, race/ethnicity was not used as a stratification factor. This contributes to cohort homogeneity.

### 4.2. Blood Collection

Whole blood was obtained by venipuncture from patients and healthy pregnant controls and collected in K_2_EDTA Vacutainer tubes (Becton Dickinson, Franklin Lakes, NJ, USA). Samples were centrifuged at 800× *g* for 15 min at 4 °C (Sigma 2-16KL, Merck, Osterode, Germany), and plasma, along with the leukocyte layer, was carefully removed. The remaining RBC fraction was washed three times with phosphate-buffered saline (PBS; 140 mM NaCl, 2.7 mM KCl, 8 mM Na_2_HPO_4_, 1 mM KH_2_PO_4_, pH 7.4) at 800× *g* for 15 min at 4 °C. Washed RBCs were finally resuspended in three volumes of PBS and used for subsequent experiments. For the aging experiment, RBCs were maintained under sterile conditions in the same buffer solution at 4 °C.

### 4.3. Sample Preparation

For AFM analysis, washed RBCs were diluted in PBS to achieve a final hematocrit of 4%. RBC smears were prepared in duplicate following the method of Dinarelli et al. [15]. In brief, 15 μL of RBC suspension was mixed 1:1 (*v*/*v*) with autologous plasma to reduce osmotic stress and maintain cell morphology, as recommended by Longo et al. [17]. The resulting suspension was gently applied onto poly-L-lysine–coated coverslips to ensure adhesion and prevent cell movement during drying and imaging. No chemical fixation was employed. After 30 min of air-drying under controlled humidity, samples were ready for AFM imaging.

Before each DSC measurement, RBCs were washed three times with PBS, and the suspension was adjusted to a hemoglobin concentration of 8 mg/mL determined spectrophotometrically.

### 4.4. DSC Measurements

DSC was performed using a DASM-4 scanning calorimeter (Biopribor, Pushchino, Russia). Scans were carried out from 30 to 90 °C at a rate of 1 °C/min. To prevent degassing, a 2 atm overpressure was maintained over the liquid in the calorimetric cells throughout the scans. The reversibility of thermal transitions was evaluated by performing a second scan immediately after cooling. As transitions were irreversible, the reheating thermogram served as the instrumental baseline. Thermograms were normalized to hemoglobin concentration, smoothed, and analyzed using OriginPro 2018. Key thermodynamic parameters, i.e., denaturation temperature (T_m_), and the excess heat capacity (c_P_^ex^) of resolved transitions were extracted from the scans.

### 4.5. Atomic Force Microscopy

RBC nanomechanical properties were assessed using an MFP-3D AFM (Asylum Research, Oxford Instruments, Santa Barbara, CA, USA) in contact mode at 22–24 °C. Silicon AFM probes (Nanosensors, qp-Bio, Neuchatel, Switzerland) with a nominal spring constant of 0.3 N/m, 50 kHz resonance frequency, and 8 nm conical tip radius were used. The cantilever stiffness was selected to be low (k < 0.4 N/m), which is appropriate for contact-mode measurements on soft biological samples, minimizing membrane deformation while enabling reliable force measurements. The small tip radius further allows high-resolution mapping of fine surface features at the nanoscale. The nominal tip radius, which corresponds to the selected low-stiffness (soft) cantilevers, was specified by the manufacturer.

Force–distance curves were acquired on approximately 25 cells per sample to ensure reproducibility and statistical robustness, with a 16 × 16 force map at a scanning speed of 3.7 µm/s.

The AFM tip was calibrated on a clean glass substrate before each session. Maximum applied forces ranged from 10 to 20 nN, avoiding nonlinear sample responses.

Apparent Young’s modulus (Ea) was determined by fitting the force–indentation curves to the Hertz–Sneddon model for a conical indenter:

$$F(\delta )\;=\;\frac{2Etan\left(\alpha \right)}{\pi \left(1-\nu^{2}\right)}\delta^{2}$$

where *F(δ*) is the applied force, *δ*—the indentation depth, *α*—the half-opening angle of the tip, and *ν*—the Poisson ratio (assumed 0.5 for RBCs). A Poisson’s ratio of 0.5 was assumed for RBCs, reflecting their near-incompressible, highly hydrated nature; this value is commonly used in AFM-based mechanical analyses of cells and corresponds to an upper limit for biological materials undergoing predominantly volume-conserving deformations.

### 4.6. Advanced Oxidation Protein Product Evaluation

The level of AOPP was estimated in blood plasma according to the protocol of Witko-Sarsat et al. [52], with additional centrifugation of the samples as suggested by Taylor et al. [53]. The absorption at 354 nm of samples diluted to a final concentration of 4.3 mg protein/mL was determined spectrophotometrically (Specord 50+Analytic, Jena, Germany), and chloramine T equivalents were determined from a standard curve.

### 4.7. Statistical Analysis

Statistical analysis was performed using linear mixed-effects models implemented in JASP (Version 0.18; JASP Team, University of Amsterdam, Amsterdam, The Netherlands). This approach was used to account for repeated measurements obtained from the same RBC samples over time and for unequal group sizes.

#### 4.7.1. Clinical and Hematological Characteristics

Normality of distribution was assessed using the Shapiro–Wilk test. As approximately 30% of the clinical and hematological variables did not meet normality assumptions, comparisons among the three groups (pregnant controls, non-severe preeclampsia, severe preeclampsia) were performed using the Kruskal–Wallis test. When significant differences were detected, post hoc pairwise comparisons were conducted using Dunn’s test with Bonferroni correction. These variables are presented as median (interquartile range). Proteinuria levels between the two preeclampsia subgroups were compared using the Mann–Whitney U test.

#### 4.7.2. Calorimetric and Nanomechanical Parameters

Thermodynamic and nanomechanical parameters obtained during RBC storage were analyzed using linear mixed-effects models to account for repeated measurements over time and unequal group sizes. Time in Days (1, 25, and 45 after RBC isolation), Group (pregnant control, non-severe preeclampsia, severe preeclampsia), and their interaction (Day × Group) were specified as fixed effects, while subject was included as a random effect to account for within-sample dependency. Linear mixed-effects modeling does not require balanced group sizes or the assumption of sphericity and is therefore appropriate for longitudinal clinical data.

For each model, F-values, degrees of freedom, *p*-values, and partial eta squared (η^2^_p_) were reported. Effect sizes were estimated using partial eta squared (η^2^_p_) and interpreted according to conventional benchmarks: small (≈0.01), medium (≈0.06), and large (≥0.14) [54]. Degrees of freedom were estimated using the Satterthwaite approximation. When significant main or interaction effects were detected, post hoc pairwise comparisons were performed using estimated marginal means with a Bonferroni adjustment for multiple comparisons. Statistical significance was set at *p* ≤ 0.05, and all tests were two-tailed.

Thermodynamic variables are presented as mean ± SD, whereas nanomechanical parameters (Young’s modulus) are presented as median (interquartile range).

## 5. Conclusions

Our results reveal that preeclampsia induces marked, severity-dependent alterations in RBCs at molecular and mechanical levels. DSC analysis demonstrated that, although spectrin destabilization primarily reflects physiological aging, Band 3 and hemoglobin exhibit significant Group × Day effects, identifying the Band 3–Hb macrocomplex as the principal site of disease-specific vulnerability. Severe PE showed earlier and more pronounced shifts in T_m_^Hb^ and T_m_^Band3^, along with reductions in c_P_^Hb^, consistent with disrupted membrane–cytoskeletal interactions and oxidative modification.

AFM measurements confirmed that these molecular alterations translate into nanomechanical consequences. Control RBCs displayed adaptive softening during aging, non-severe PE retained partial remodeling capacity, and severe PE exhibited pathological stiffening and specific mechanical alterations upon prolonged storage. Linear mixed-effects modeling revealed strong time, group, and interaction effects for Young’s modulus, in line with the thermodynamic parameters remodeling.

Together, the combined DSC and AFM approach demonstrates that severe preeclampsia induces selective destabilization of the Band 3–hemoglobin macrocomplex, linking protein instability to impaired mechanical integrity and suggesting a biophysical basis for increased RBC fragility and microcirculatory dysfunction.

From a translational perspective, the combined thermodynamic–nanomechanical profiling approach may provide sensitive biophysical markers for identifying early subclinical RBC damage not detectable by routine hematological tests. Future studies should focus on the application of advanced mathematical and multivariate modeling of integrated datasets encompassing both non-severe and severe PE, to identify novel interrelationships and potential diagnostic markers. In addition, integrating cellular and plasma biophysical parameters into a unified composite disease index (a cellular biophysical “score”) may offer a more comprehensive and quantitative framework for disease stratification and risk assessment. Such approaches could enhance the predictive and diagnostic value of biophysical profiling in preeclampsia and support its future clinical translation.

## Figures and Tables

**Table 1:** Clinical characteristics and hematological parameters derived for pregnant controls (PC) and women with non-severe and severe PE. Data are presented as median (Q1; Q3). Proteinuria values are presented as mean ± SD. Overall group comparisons were performed using the Kruskal–Wallis test followed by Dunn–Bonferroni post hoc analysis. Different superscript letters (a–c) within a row indicate statistically significant differences between groups (*p* ≤ 0.05). ^†^ Proteinuria was compared between PE groups using the Mann–Whitney U test, where *m* indicates values determined only for a limited number of patients for which data were available. Mean values for proteinuria were calculated using *m* values only.

Characteristic	Reference Values for Pregnant Women	PC (*n* = 14)	Non-Severe PE (*n* = 20)	Severe PE (*n* = 8)	*p*
Maternal age (years)		28 (25; 32.8) ^a^	30 (27; 34) ^ab^	35 (31.8; 36.3) ^b^	0.029
Mean BP (systolic)		115 (114; 120) ^a^	145 (135; 148) ^b^	162 (160; 167) ^b^	<0.001
Mean BP (diastolic)		75 (65; 76) ^a^	94 (90; 95) ^b^	105 (90; 111) ^c^	<0.001
GA at delivery		39 (38; 40) ^a^	37 (31.8; 38) ^b^	29 (28; 29.5) ^c^	<0.001
Newborn weight (g)		3330 (3155; 3457.5) ^a^	2770 (2130; 3080) ^b^	1060 (852.5; 1425) ^c^	<0.001
Proteinuria (mg in 24 h urine collection) ^†^		-	482 ± 320 (*m* = 8)	1736.6 ± 320 (*m* = 6)	0.041
RBC count (T/L)	4.0–6.2	3.99 (3.81; 4.2)	3.87 (3.54; 4.16)	3.8 (3.65; 4.29)	0.197
Hb (g/L)	120–160	120 (113.5; 125)	120 (111; 127)	125 (118.5; 128)	0.924
Ht (L/L)	0.36–0.54	0.36 (0.35; 0.37)	0.37 (0.33; 0.38)	0.37 (0.36; 0.39)	0.818
MCV (fL)	82–100	92.15 (87.6; 96.35)	93.3 (87.7; 97)	96.9 (90.5; 98.95)	0.938
MCH (pg)	29.0–45.0	31.4 (29.2; 32.55)	30.1 (29.6; 33.1)	32.7 (30.15; 33.15)	0.877
MCHC (g/L)	240–360	336 (332.5; 341)	332 (329; 339)	335 (332; 336)	0.976
RDW (%)	11.5–14.5	13.8 (13; 14.5)	13.8 (12.9; 15.4)	12.5 (11; 14.3)	0.226

**Table 2:** Thermodynamic parameters (transition temperature of spectrin (T_m_^spectrin^), Bands 2.1, 4.1 and 4.2 (T_m_^Band2–4^), Band 3 protein (T_m_^Band3^), and Hb (T_m_^Hb^), and the heat capacity of Band 3 protein (c_P_^Band3^) and Hb (c_P_^Hb^)), measured in the process of erythrocyte aging for the period 1–45 days after isolation of erythrocytes from pregnant women in the third trimester of pregnancy and women with non-severe and severe preeclampsia. Lowercase letters indicate statistically significant differences between storage days within the same group, whereas uppercase letters indicate differences between groups within the same day, according to post hoc pairwise comparisons with Bonferroni adjustment (*p* < 0.05).

Parameter	Group	Day 1	Day 25	Day 45
T_m_^spectrin^ (°C)	PC	50.4 ± 0.2 ^a^	50.0 ± 0.3 ^aA^	48.6 ± 0.4 ^b^
	non-severe PE	49.9 ± 0.3 ^a^	49.1 ± 0.3 ^aB^	48.7 ± 0.2 ^a^
	severe PE	49.9 ± 0.2 ^a^	50.3 ± 0.2 ^aA^	49.0 ± 0.2 ^b^
T_m_^Band2–4^ (°C)	PC	57.7 ± 0.3 ^A^	57.3 ± 0.16	57.9 ± 0.29 ^A^
	non-severe PE	56.7 ± 0.2 ^bB^	57.6 ± 0.17 ^a^	57.8 ± 0.24 ^aA^
	severe PE	57.8 ± 0.2 ^aA^	57.3 ± 0.2 ^a^	56.3 ± 0.19 ^aB^
T_m_^Band3^ (°C)	PC	63.3 ± 0.19 ^aA^	63.0 ± 0.13 ^aA^	62.83 ± 0.11 ^aA^
	non-severe PE	63.3 ± 0.16 ^aA^	62.4 ± 0.09 ^bB^	61.9 ± 0.12 ^cB^
	severe PE	62.8 ± 0.08 ^aB^	62.2 ± 0.09 ^bB^	62.10 ± 0.12 ^bAB^
c_P_^Band3^ (J/kg⋅deg)	PC	167.4 ± 7.5 ^aA^	171.5 ± 13.4 ^aA^	167.4 ± 15.5 ^aA^
	non-severe PE	159.0 ± 6.7 ^aA^	121.3 ± 4.2 ^bB^	92.0 ± 5.0 ^cB^
	severe PE	171.5 ± 8.0 ^aA^	117.2 ± 7.5 ^bB^	301.2 ± 15.5 ^cC^
T_m_^Hb^ (°C)	PC	71.9 ± 0.16 ^aA^	71.6 ± 0.18 ^abA^	71.2 ± 0.21 ^bA^
	non-severe PE	72.0 ± 0.17 ^aB^	71.5 ± 0.16 ^abA^	70.9 ± 0.25 ^bB^
	severe PE	71.9 ± 0.35 ^aA^	70.8 ± 0.29 ^bB^	69.6 ± 0.32 ^cC^
c_P_^Hb^ (J/kg⋅deg)	PC	3054.3 ± 753.1 ^aA^	2677.8 ± 753.2 ^bA^	2133.8 ± 669.4 ^cA^
	non-severe PE	2970.6 ± 502.1 ^aB^	2384.9 ± 753.1 ^bB^	1924.6 ± 753.1 ^cB^
	severe PE	2928.8 ± 376.6 ^aB^	2384.9 ± 878.6 ^bB^	1757.3 ± 711.3 ^cB^

**Table 3:** Linear mixed-effects model analysis of the effects of storage time (Day), disease severity (Group), and their interaction (Day × Group) on calorimetric parameters of major erythrocyte proteins. Degrees of freedom (df), F-values (F), *p*-values (*p*), and partial eta squared (η^2^_p_) are presented for the investigated effects.

Parameter	Factor	df	F	*p*	η^2^_p_
T_m_^spectrin^	Day	2, 117	120.4	<0.001	0.673
	Group	2, 58	8.07	<0.001	0.218
	Day × Group	4, 117	0.647	0.630	0.022
T_m_^Band2–4^	Day	2, 117	5.32	0.006	0.083
	Group	2, 58	3.15	0.049	0.098
	Day × Group	4, 117	1.12	0.352	0.037
T_m_^Band3^	Day	2, 117	15.6	<0.001	0.210
	Group	2, 58	12.1	<0.001	0.294
	Day × Group	4, 117	3.82	0.007	0.115
c_P_^Band3^	Day	2, 117	48.3	<0.001	0.452
	Group	2, 58	11.7	<0.001	0.287
	Day × Group	4, 117	7.21	<0.001	0.198
T_m_^Hb^	Day	2, 117	33.4	<0.001	0.364
	Group	2, 58	9.86	<0.001	0.253
	Day × Group	4, 117	5.14	0.001	0.150
c_P_^Hb^	Day	2, 117	27.6	<0.001	0.320
	Group	2, 58	7.42	0.001	0.204
	Day × Group	4, 117	4.83	0.002	0.142

**Table 4:** Median (Q1; Q3; 95% CI) Young’s modulus values of RBCs from controls, non-severe PE, and severe PE, measured on day 1 and after 25 and 45 days of storage. Lowercase letters indicate differences between storage days within the same group, whereas uppercase letters indicate differences between groups within the same day of storage.

Group	Ea (MPa)		
	Day 1	Day 25	Day 45
PC	1.46 (1.38; 1.8; 1.22–1.71) ^aA^	1.03 (0.95; 1.11; 0.83–1.25) ^bA^	0.95 (0.91; 1.01; 0.86–1.27) ^bA^
Non-severe PE	1.64 (1.49; 1.71; 1.24–1.58) ^aB^	1.31 (1.24; 1.38; 1.15–1.46) ^bB^	1.27 (1.22; 1.34; 1.16–1.44) ^bB^
Severe PE	1.81 (1.38; 1.8; 1.35–2.40) ^aC^	1.63 (1.53; 1.73; 1.31–1.98) ^bC^	2.43 (2.31; 2.51; 1.24–2.75) ^cC^

**Table 5:** Linear mixed-effects model analysis of the effects of storage time (Day), disease severity (Group), and their interaction (Day × Group) on Young’s modulus values of RBCs. Degrees of freedom (df), F-values (F), *p*-values (*p*), and partial eta squared (η^2^_p_) are presented for investigated effects.

Parameter	Factor	df	F	*p*	η^2^_p_
Young’s modulus	Day	2, 1468	4966	<0.001	0.871
	Group	2, 2799	1387	<0.001	0.498
	Day × Group	4, 2792	1877	<0.001	0.729

**Table 6:** Advanced oxidation protein products (AOPP) values for blood plasma from normotensive pregnant controls (PC), non-severe PE, and severe PE. Values are presented as mean ± SD. *p*-values indicate statistical significance compared to the control group, evaluated using the Mann–Whitney U test.

Group	AOPP (μM Chloramine T Equivalents)	*p*
PC	134.18 ± 30.8	
non-severe PE	151.06 ± 45.3	0.53
severe PE	308.70 ± 51.5	0.03

## Data Availability

The original contributions presented in this study are included in the article. Further inquiries can be directed to the corresponding authors.

## Abbreviations

The following abbreviations are used in this manuscript:

PE	Preeclampsia
RBC	Red blood cell
DSC	Differential scanning calorimetry
AFM	Atomic force microscopy
MCV	Mean corpuscular volume
MCH	Mean corpuscular hemoglobin
MCHC	Mean corpuscular hemoglobin concentration
Hb	Hemoglobin
Ht	Hematocrit
df	Degrees of freedom
CAT	Catalase
